# Exploring the lived experience: impact of dementia diagnosis on individuals with cognitive impairment - a qualitative study

**DOI:** 10.1186/s12877-024-04665-3

**Published:** 2024-02-01

**Authors:** Inger Molvik, Grete Kjelvik, Geir Selbæk, Anne Marie Mork Rokstad

**Affiliations:** 1 The Norwegian National Centre for Ageing and Health, Vestfold Hospital Trust, Tønsberg, Norway; 2 Institute of Clinical Medicine, University of Oslo, Oslo, Norway; 3 Department of Geriatric Medicine, Oslo University Hospital, Oslo, Norway; 4 Faculty of Health Sciences and Social Care, Molde University College, Molde, Norway; 5 Ageing and Health, Postboks 2136, Tønsberg, 3103 Norway

**Keywords:** Cognitive impairment, Experience, Older adults, Dementia diagnosis, Timely diagnosis, Qualitative study

## Abstract

**Objective:**

Although knowledge about the experience of being diagnosed with dementia is limited, with the expected rise in dementia’s prevalence in the coming decades, such knowledge is pivotal for the people diagnosed, their families, and healthcare planners. Thus, the aim of our study was to explore the experience of living with cognitive impairment and dementia and the impact of being diagnosed with dementia.

**Method:**

A qualitative design was applied. Participants were recruited based on age-adjusted values below ​​threshold values on the Montreal Cognitive Assessment Scale (i.e. 70–79 years, < 22; 80–89 years, < 21; 90 + years, < 20), and the sample ultimately included 15 participants: six with and nine without a documented dementia diagnosis. Qualitative content analysis was performed on the transcribed interviews in four steps to identify codes, categories, and the overall theme.

**Results:**

Three major categories emerged from the interviews: (1) experiences with changes, (2) experiences with being diagnosed with dementia, and (3) existential experience. All participants with and most participants without a dementia diagnosis experienced changes in cognition.

**Conclusion:**

Our findings imply that being diagnosed with dementia is a relief because it explains observed cognitive and functional decreases and reduces confusion, shame and stigma. However, it also raises concerns about an unknown future. Most participants not diagnosed with dementia reported having little or no difficulty with everyday living and leading a fulfilling life. Those findings emphasise the significance of timely versus early diagnosis.

## Introduction

Despite the projected increase in the prevalence of dementia in the coming decades [1–3], it is also commonly accepted that dementia is underdiagnosed in many low- and middle-income countries and that diagnoses are often made several years after the disease’s onset [4]. Such underdiagnosis can be expected to make it difficult for people with dementia to understand what is wrong and to obtain relevant information about their conditions [5]. According to the Norwegian national guideline on dementia [6], a diagnosis is essential for offering personalized information to individuals and their relatives. When cognitive impairment begins affecting daily life, a diagnosis becomes crucial for accessing appropriate support and follow-up, aligning with international guidance. Obtaining an early dementia diagnosis not only provides access to support and resources for both individuals and their networks but also improves overall quality of life and aids in future planning [7].

Several studies on the experience of living with dementia have increased the awareness of the reality that people with dementia can communicate their own needs, desires, and worries [8–10]. Discovering and addressing the needs of people with dementia are critical endeavours, because unmet needs can reduce quality of life, increase discontent, and drive a sense of despair [11–13]. In turn, studies describing subjective experiences of living with dementia have summarised that people with dementia need to be accepted and appreciated in order to be able to develop effective coping mechanisms for daily life and gain perspective on their circumstances [14–17].

According to previous research, being diagnosed with dementia can significantly influence a person’s life and result in negative outcomes such as worry and anxiety [18–20], post-traumatic stress [21], and even suicidal ideation [22]. Nevertheless, other studies have suggested that being diagnosed with dementia can also provide psychological relief [19, 23] and/or promote healthy behaviours [24].

In the context of dementia, *timely diagnosis* is defined as «access to accurate diagnosis at a time in the disease process when it can be of most benefit to them (i.e., people with dementia and families)» [25]. In recent years, timely diagnosis has been emphasised as being more person-centred and respectful of individual rights than early diagnosis. Although early diagnosis may be favourable for accessing resources and support, as well as for preparing for the future, studies have suggested that timely diagnosis better accommodates the readiness and ability of people with dementia and those around them to benefit from the diagnosis [26, 27]. Still others have shown that early diagnosis provides time for everyone affected to adjust while the patient is can still actively participate and affords earlier access to guidance, financial support, and pharmacological and non-pharmacological treatments [28].

To date, research on the experience of being diagnosed with dementia versus the impact of not being diagnosed with dementia while living with cognitive impairment compatible with dementia has been limited. Moreover, to gain access to those experiences, many studies have depended on information from caregivers, not people with dementia [29, 30]. However, exploring those individuals’ subjective experiences is important for planning services to be offered to them and their families.

Against that background, the aim of our study was to explore the experience of living with cognitive impairment compatible with a possible dementia and the impact of being diagnosed with dementia.

## Materials and methods

A qualitative descriptive design using individual interviews was followed to access the experiences of people living with cognitive impairment.

### Participants and recruitment

Participants were recruited from the Trøndelag Health Study (HUNT; [31], a longitudinal population health study in Norway, and the sub-study HUNT4 70+, which was completed in 2019. The significance of the HUNT Study gives thorough knowledge on health-related lifestyle, disease prevalence and genotypic associations [32]. A comprehensive dataset incorporating questionnaires, clinical measurements, and biological samples. Data from the cognitive tests in the sub-study HUNT 4 70 + formed the basis for further recruitment to this particular study. The total number of participants in HUNT4 70 + was 11,700, and all survivors were invited to participate in a follow-up study, Ageing in Trøndelag (AiT), in the autumn of 2021, four years after the baseline assessment.

Participants from four municipalities in Trøndelag were recruited while at testing stations during data collection for the AiT study. The staff at the testing stations distributed information about the study, an invitation to participate in individual interviews, and the research group’s contact information. All individuals interested in participating were contacted by the research group.

The selection of participants was random and based on age-adjusted threshold values ​​on the Montreal Cognitive Assessment Scale (MoCA; [33] at the time of data collection for the AiT study. A brief screening tool that provides an immediate assessment of a person’s cognitive condition, MoCA is a well-known, widely used instrument for identifying and diagnosing cognitive problems, particularly in older adults. The age-specific cut-off scores ​on MoCA used to identify cognitive impairment were < 22 for 70–79-year-olds, < 21 for 80–89-year-olds, and < 20 for individuals 90 years old or older (ref MoCA).

Variation in the sample was ensured by including both women and men 72–84 years old with a mild to moderate degree of cognitive impairment. All participants were either living alone or with a partner (i.e. cohabitant), and the sample included participants from urban and rural municipalities. The participants’ characteristics are presented in Tables 1 and 2.

**Table 1:** Participants diagnosed with dementia

Name	Civil status	Age (in years)	Gender		Urban or rural residency	MoCA score
Doris	Married	76	Woman		Urban	10
Dagny	Married	79	Woman		Rural	17
Diana	Married	77	Woman		Urban	20
Daniel	Married	83	Man		Rural	18
David	Married	84	Man		Urban	8
Dennis	Married	80	Man		Urban	12

*MoCA: Range: 8–20, mean = 14*

*Age: Range: 76–84, mean = 80*

**Table 2:** Participants not diagnosed with dementia

Name	Civil status	Age (in years)	Gender	Urban or rural residency	MoCA score
Oscar	Cohabitant	72	Man	Rural	20
Olivia	Single	77	Woman	Urban	19
Odin	Married and living alone	84	Man	Urban	17
Oline	Single	81	Woman	Urban	11
Oda	Single	77	Woman	Urban	15
Oliver	Cohabitant	75	Man	Urban	17
Olav	Married	79	Man	Rural	17
Olga	Married	76	Woman	Rural	20
Otto	Married	76	Man	Rural	21

MoCA: range: 11–21, mean = 17

Age: range: 72–84, mean = 77

### Preunderstanding

The first author (IM) has a master’s degree in gerontology and extensive experience with working in nursing homes and with people with dementia and their next of kin. The three co-authors (GK, GS and AMMR) are medical and health and social science researchers, as well as one registered nurse (AMMR) and one psychiatrist (GS). They have a wealth of experience in the subject of dementia.

### Interviews

The interviews were conducted by IM, with some assistance from AMMR, using a semi-structured interview guide.

The questions focused on experiences with living with cognitive impairment, particularly regarding changes in cognition, function, and quality of life, as well as experiences related to having been diagnosed with dementia. The opening questions were introduced by repeating the aim of the study and a reminder of their acceptance to participate.

The interviews were performed either at the AiT testing station, in a meeting room in a municipal office, or at the participants’ homes based on the participants’ preferences in the period between November 2021 and August 2022. Before questions were asked, the aim of the study was reiterated, and the participants were reminded of their acceptance to participate. The interviews lasted between 30 and 60 min, were audio-recorded, and were transcribed verbatim before analysis.

### Analysis

The transcribed interviews were analysed with reference to Malterud [34] using systematic text condensation in four steps. First, all transcripts were read to gain an overall impression and identify preliminary themes that could meet the aim of the study. Second, meaning units in the text were extracted, labelled with codes, and categorised. Third, the preliminary themes were converted into code groups and subgroups according to the meaning unit. Fourth and finally, a summary of the findings of the interview analysis was prepared.

## Results

The sample comprised 15 participants: six who reported being diagnosed with dementia and nine who had cognitive impairment compatible with possible dementia but who had not been diagnosed with dementia, at least according to them. Those two groups are hereafter denoted as «living with a diagnosis» and «living without a diagnosis», respectively. All participants had a MoCA score in accordance with the inclusion criteria.

The characteristics of the study population are shown in Tables 1 and 2.

As shown in Table 3, the analysis revealed three categories of experiences with living with cognitive impairment with or without a dementia diagnosis: (1) experiences with changes, (2) experiences with being diagnosed with dementia, and (3) existential experience.

**Table 3:** Experiences with living with cognitive impairment and dementia

Category	Experiences with changes	Experiences with being diagnosed with dementia	Existential experiences
*Subcategories*	Changes in cognition Changes in function Emotional reactions	Reactions upon receiving the diagnosis Transparency and stigma	Relations Dignity and future

### Experiences with changes

The category «Experiences with changes» is divided into three subcategories, all capturing the participants’ experiences: (1) changes in cognition, (2) changes in function, and (3) emotional reactions.

#### Changes in cognition

Changes in cognition led to several challenges reported by participants living with a diagnosis, including the experience of becoming more forgetful and finding it harder to express themselves: «I find it more difficult to remember things and to orientate myself» (Doris). Some experienced saying things that were not true, and some felt more tired than before being diagnosed. They had become more sensitive to stress and explained that they needed more rest in order to concentrate. Doris was also concerned about her need for predictability and for more time to prepare before meeting other people. Diana found it painful to experience not remembering where she was going when she and her husband went for a drive. She described experiencing «a complete stop» in her mind: «Sometimes I don’t know what I am supposed to do. It comes to a complete stop» (Diana).

Most participants living without a diagnosis also experienced changes in cognition. They found it particularly difficult to remember names but also reported more serious symptoms such as misplacing things, being forgetful, and struggling to learn new things: «I have a hard time learning new things» (Oline). Otto found it challenging to perceive the content of newspapers and reported having to read articles repeatedly in order to comprehend them: «I have to read something many times to comprehend it».

Some living without a diagnosis found such changes to be unproblematic: «I’m healthy, and there’s no need to talk to the doctor about it» (Oscar). Others, however, had considered discussing the changes with their general practitioner (GP) and being assessed for cognitive impairment. Olivia was unsure whether she had a diagnosis of dementia after her brain had been scanned and she found herself experiencing extreme forgetfulness: «I don’t know if I have dementia, but I am very forgetful». Otto and Olav were unsure whether a stroke had caused the changes that they had experienced, which included dizziness as well as problems with speaking and/or writing. However, most participants living without a diagnosis had not sought help for their symptoms but said that they would contact their GP if they experienced more severe problems: «If my memory gets worse, I’ll have to go to the GP» (Olivia).

#### Changes in function

For some, changes in function had limited their driving capabilities, and half of the participants living with a diagnosis had lost their driver’s licence, which they viewed as being both unfair and difficult. In fact, Daniel said that losing his driver’s licence was the worst part of being diagnosed with dementia: «The worst part was that I wasn’t allowed to drive a car anymore». Others understood that driving was no longer relevant, whereas ones who still had their driver’s licences were grateful that they could continue to drive. None of the participants living without a diagnosis had lost their driver’s licences, and many of them still drove and considered themselves to have mastery in the skill. However, Oda, living without a diagnosis, reported becoming calmer after taking the initiative to no longer drive.

Considering other functions, several participants living with a diagnosis reported still being capable of doing most activities of daily living, including showering, making coffee, cleaning, and shopping: «I manage to shower and make coffee» (David). Several also claimed that their functional capacity was reasonably good and that they had not yet experienced major difficulties, although some had experienced limitations, including not being able to go out alone anymore. Diana described how cleaning her house had become difficult and that she lacked the initiative to face the challenge. As a result, she no longer felt as though she was a proper housewife and stated that asking for help with cleaning made her feel stupid: «I’m not a proper housewife anymore. I can’t do it» (Diana). At the same time, she described her house as no longer being suitable housing because she could not tend the garden in the way that she had done previously. Although she was ready to move into a more suitable home, thinking about the decision to move was exhausting for her. Another participant living with a diagnosis, Dagny, described wanting to be more physically active but was restricted by the fact that she was no longer allowed to go out alone: «I want to exercise more, but my husband wants to know where I am at all times».

All participants living without a diagnosis experienced coping quite well with everyday life. For example, they claimed to remember appointments and to not get lost: «I don’t forget appointments and things like that» (Olga). They also claimed to have few problems, if any, in their daily living: «I don’t have any problems now, but I don’t know what it will be like in the future» (Oliver). Several felt that they were fit and still had a lot of desire to contribute: «I haven’t lost my desire to work» (Otto).

At the time of the interviews, two participants living with a diagnosis were receiving regular municipal services: one for drug administration, the other for weekly day care at a day care centre. A participant not living a diagnosis was also receiving drug administration services from the municipality.

#### Emotional reactions

Some participants living with a diagnosis reported being irritated and angry as well as sad more often than before being diagnosed. Some were concerned that their anger and irritability were afflicting their spouses: «I take it out on my husband when I get angry» (Doris). Dagny became stressed and irritated when she could not remember people’s names and expressed sadness in having become so forgetful that she could no longer be responsible and lead activities as before: «I’m too forgetful to be responsible now. That’s a source of sadness for me» (Dagny). Diana believed that dementia had made her more nervous, while Doris experienced becoming more suspicious when meeting people and was afraid that they were talking about her behind her back: «I imagine they’re talking behind my back». Last, Otto, one of the participants living without a diagnosis, also reported being more irritated than previously: «I get annoyed more often than before» (Otto).

### Experiences with being diagnosed with dementia

The category «Experiences with being diagnosed with dementia» encompasses the experience of initially being diagnosed with dementia, namely in two subcategories: (1) reactions upon receiving the diagnosis and (2) transparency and stigma.

#### Reactions upon receiving the diagnosis

Every participant living with a diagnosis had felt relieved upon being diagnosed: «I think it was a relief to get the diagnosis» (David). It seemed crucial to finally have a name for the changes that they were experiencing and an explanation for those changes: «In receiving the diagnosis, I got a name for it [my condition] that I can share with others» (Doris). Some experienced a sense of calmness after being diagnosed. Most had expected the diagnosis upon receiving it. David was surprised upon receiving the diagnosis but had nevertheless suspected that something was not right.

Daniel experienced sadness upon being diagnosed. Although receiving the diagnosis was a relief and provided an explanation for the changes that he was experiencing, he found it difficult to accept the diagnosis: «I don’t want to admit my dementia» (Daniel). Even so, he and the others had all come to terms with being diagnosed with dementia. Although most participants had noticed a change in their cognition, a few reported becoming aware only once their family members had reacted to changes in their cognition: «My wife noticed there was a change, that I’d become slower in doing various things» (David).

#### Transparency and stigma

Most participants diagnosed with dementia were open about their diagnosis: «I’ve come out about it, even though it’s no fun to have been given that label [Alzheimer’s]» (Doris). However, some had only told their next-of-kin about the diagnosis, while others only disclosed their diagnosis upon being asked directly about their situation. Meanwhile, one preferred to describe how she felt rather than use the word *dementia*. In any case, all participants living with a diagnosis reported receiving highly understanding reactions from others when they were open about how they felt. One said that she was proud to be open about her diagnosis and refused to be ashamed of the disease. Nevertheless, Dagny reported receiving foolish answers when she was open about her diagnosis and that others wanted to diminish the symptoms: «I often get foolish answers when I tell them how I feel».

### Existential experiences

The category «Existential experiences» is divided into two subcategories: (1) relations and (2) dignity and future.

#### Relations

Of the participants living with a diagnosis, several experienced being isolated and in less contact with people than before being diagnosed: «I miss being with people» (Diana). Doris noticed having become withdrawn and quiet in social settings. She did not consider meeting people to be much fun anymore, and she often declined invitations, which marked a significant change from her accustomed behaviour. She added that she had become more tired having people around her (Doris).

Several expressed dependencies on their spouses and believed that living at home would be difficult without their support and help: «I wouldn’t be able to stay at home without my husband» (Diana). At the same time, Doris wondered how her spouse could bear living with her: «How can he live with me?» Doris also hoped that she had taught her children how to take care of her in anticipation that her condition would worsen.

Meanwhile, of participants living without a diagnosis, several experienced being more isolated and not as active anymore. Oda reported having «less contact with people now than previously» and not being in contact with friends or neighbours: «We don’t see so many people anymore». Olivia felt very lonely and said that becoming old was worse than she had imagined: «I knew that getting old was going to take its toll, but that it was going to be so damn hard? I wasn’t aware of that». She had no one to turn to if she needed help or support. She missed being out among people, going to cafés, and being able to chat with others. She was also the only participant who was open to being assigned an activity friend for more social contact:^1^ «I’ve tried phone friend, but it didn’t work. I’m open to trying an activity friend» (Olivia). By contrast, Otto was thankful that he had many people, including several good friends and friendly neighbours, who would step in if he needed help.

#### Dignity and future

Many participants living with the diagnosis were afraid of losing their dignity, and one expressed that it was important for her to participate, to be able to contribute, and to be something to someone: «It’s important for me that someone needs me» (Dagny). Doris highlighted that she was more than just her dementia and had a strong desire to live and to exercise influence over her life: «I’m not just my disease. I’m also myself sometimes».

Several were reluctant to think about the future and unsure of what it would bring. They were willing to move into a nursing home if necessary: «I know I’m getting worse, and obviously I’ll have to go to a nursing home if I get a lot worse» (Doris). Others were not afraid of the future or the disease but hoped to be able to continue living as they were at the time of the interviews for as long as possible: «I hope that I can live like this for a long time» (David). Even so, another was prepared for illnesses that come with such an advanced age: «I’m so old, and there will be more illness and ailments» (Dennis).

All but one participant living without a diagnosis said that they had a good life and would not have it any other way: «I miss nothing» (Oscar). While most did not fear the future or think about it very much, Olivia was rather anxious about what the future would bring: «I don’t dare think about the future».

## Discussion

The aim of our study was to explore the experiences of people living with cognitive impairment compatible with possible dementia and the impact of being diagnosed with dementia.

According to our findings, being diagnosed with dementia seemed to impact the participants’ experiences in everyday life. All participants living with and most participants living without a diagnosis experienced changes in cognition. The participants living without a diagnosis, however, seemed to describe the changes as being largely unproblematic and described their lives as being mostly comfortable and involving few problems, if any. All participants living with a diagnosis felt relieved upon being diagnosed and preferred to be open about their disease. Common in both groups was the experience of increased social isolation and being less active than previously. Unlike participants living without a diagnosis, many living with a diagnosis were concerned about losing their dignity and were reluctant to face an uncertain future.

### Living conditions

The contrasts between the groups can be interpreted according to differences in their living conditions. No participant living with a diagnosis lived alone, for they were all married or lived with someone, whereas two participants living without a diagnosis lived alone. That circumstance may align with past findings that older people who live alone tend to be diagnosed with dementia later than older adults who live with at least one other person [19, 37].

### The relief of being diagnosed

A major finding was that all participants diagnosed with dementia had felt relieved upon being diagnosed. Some viewed it as bringing clarity to their situations, inviting self-acceptance, and making spouses, relatives, and friends more understanding of their conditions. That finding is supported by findings from past studies, in which participants reported feeling validated by their diagnosis because their partners had frequently misinterpreted their forgetfulness as a sign of apathy. Being diagnosed also validated the participants’ concerns, sated their curiosity, and prompted them and their families to take action to modify expectations and future plans [19, 28, 38]. Studies on Mild Cognitive Impairment (MCI) have also confirmed that being diagnosed is preferable to not knowing what causes cognitive changes [39, 40]. Several participants in our study reported feeling calm after being diagnosed, which is consistent with the findings of Carpenter et al. [23], who discovered that anxiety symptoms appear to wane after they received diagnostic feedback. Individuals who were anxious at the start of the examination procedure were more likely to feel a wave of relief once they received diagnostic information.

### Openness about the diagnosis

The majority of the participants were open about their diagnosis, although a few chose to disclose it exclusively to family and friends. On that count, information and mass media initiatives can assist with minimising stigma by altering perceptions of and attitudes about dementia in the general public and among healthcare professionals. People living with dementia might benefit from adopting a more positive, constructive attitude towards their diagnosis, which can lead to increased well-being and quality of life [41]. Dagny disliked the term *dementia* and preferred to explain her symptoms instead of the diagnosis, while several others told only their family members and closest friends. Fear of losing one’s identity in the eyes of others commonly inhibits people from disclosing their dementia diagnosis, which prevents them from benefitting from enhanced quality of life, better future planning, and a positive support network that can promote independence [42, 43]. O’Connor et al. [44] have emphasised the significance of being aware of stigmatising and discriminatory attitudes regarding a dementia diagnosis, whereas openness can help people with dementia to avoid feelings of guilt and shame about their disease.

Several participants living with a diagnosis were concerned about losing their dignity and control over their lives and underscored the need to be able to contribute and to be needed, as well as to retain a sense of still being oneself and not simply a disease. Several participants were also eager to emphasise how much they could still manage and how they still felt a sense of self. According to the findings of a recent systematic review, interactions with others alter people’s sense of self and may double as an empowering means of support but can also contribute to feelings of marginalisation and isolation [45].

Having a sense of control over one’s life can provide support and autonomy among people with dementia and thereby potentially delay the need for care [46]. A positive approach can be defined as pursuing the desire to live well, not allowing dementia to take over one’s life, and maintaining hopes and aspirations regardless of future prospects [14]. Such a positive approach was described by Dennis who expressed gratitude for his long and happy life and for having attained such an advanced age, albeit with a realistic expectation that old age also presents new challenges.

### Changes in social relationships

All participants living with or without a diagnosis recognised challenges caused by changes in cognition and function. Participating in social events and meaningful activities had become more challenging for participants living with a diagnosis. As a result, some felt inept and unable to participate as much as they would like in the household and in society, among other settings. That finding is consistent with the findings of Bjørkløf et al. [14], who also found that a lack of social contact and meaningful activities leads to loneliness, isolation, emptiness, and/or boredom. In both groups, many participants reported being more isolated and having less contact with people than previously. As in depression, social isolation may be a symptom or aspect of dementia [21, 47, 48]. By contrast, social changes in dementia are less recognised as symptoms of the disease by both caregivers and patients. However, when dementia is not acknowledged as a factor behind social changes, more negative consequences, including social isolation, can occur [49]. Along similar lines, [50] have suggested that being diagnosed with dementia may have unintended effects for social relationships, such as decreases in social activities.

### Timely diagnosis

A discrepancy in the participants’ experiences with managing in everyday life, as well as their feelings and views about the future, warrants consideration. Whereas several participants living with a diagnosis feared the future, most participants living without a diagnosis chose not to worry. Some living with a diagnosis found the future to be so frightening and unpredictable that they avoided thinking about it altogether. Other studies have shown similar results, including that people with dementia feel apprehensive about the future [14, 42]. In our study, all but one participant living without a diagnosis claimed that they were happy with their life and did not wish to change anything. Even so, ignorance or avoidance of a dementia diagnosis might be an adequate coping strategy for maintaining one’s identity and quality of life [14, 51]. Self-presentation strategies that imply a lack of awareness in people with dementia may be regarded as responses to their social circumstances in order to avoid experiencing stigma and generating stigma towards dementia in general [52].

The subjective experience of cognitive decline prior to being diagnosed with dementia determines how one responds to the diagnosis [42]. Although all participants in our study living with a diagnosis experienced relief upon being diagnosed, that outcome could have been due to experiences with cognitive and functional changes. Moreover, several had expected to be diagnosed. That possibility suggests that the diagnosis was made in a timely rather than too early [25–27]. Only two participants living with a diagnosis were regularly receiving municipal services at the time of their interviews, which may reflect the absence of major functional and psychological problems in their everyday lives.

### Strength and limitations

To our knowledge, our study was the first to include people with significant diagnosed and undiagnosed cognitive impairment. Even though the sample had only 15 participants, the data revealed some interesting contrasts in how the two groups shared their experiences. The information about whether or not the participants had received a dementia diagnosis was based on self-report. That circumstance might imply that some of the stated undiagnosed might have been diagnosed but were not aware of it or did not remember being diagnosed. This is a limitation that needs to be considered when interpreting the results. Obtaining information from the next of kin could have confirmed or refuted additional diagnostic information. However, our study’s aim was to explore the participants’ subjective experiences of having received a diagnosis or not. Furthermore, the congruence of the same average age and MoCA scores in both groups would have strengthen the results. However, in our dataset, the group diagnosed with dementia exhibits both a higher score and a higher average age compared to the group without a diagnosis. The first author’s work experience with people with dementia and their next of kin may have contributed to the considerable amount of data material in need of thorough interpretation, which complicated analysis as well as how the results have been presented. Based on this, and to ensure trustworthiness and reflexibility, two of the co-authors (GK, and AMMR) took part in the analyses and interpretation of the data. The first author’s rather modest interview expertise was another possible limitation; however, one co-author (AMMR) has substantial interview experience with the patient population and took part in the first four interviews to supervise the first author to enhance the reliability and credibility of the findings.

## Conclusion

Our findings reveal that being diagnosed with dementia is a relief when it explains observed cognitive and functional declines. The diagnosis provides a reason for the changes experienced, which reduces confusion, embarrassment, and stigma. At the same time, it also heightens anxiety about the uncertainty of the future. Even so, participants who had been diagnosed with dementia had accepted their diagnosis, which allowed them to be more open about their challenges. Of course, living with cognitive impairment without being diagnosed with dementia might also have advantages. While several participants living with a diagnosis were concerned about their loved ones and were afraid of losing themselves, most participants living without a diagnosis reported experiencing few to no challenges in everyday life, being unconcerned about the future, and living good lives. Those findings emphasise the significance of timely versus early diagnosis. To further validate our findings, it would be useful to investigate how next of kin perceive the diagnosis of dementia in their family members.

## Data Availability

The data used or analysed during the current study are available from the corresponding author on reasonable request.

## Footnotes

1 The Visitor Service is the Red Cross’s oldest and largest social care activity [35]. The Norwegian Red Cross. This is the Red Cross 2016 [Available from: https://www.rodekors.no/globalassets/_om-rode-kors/sentrale-lover-og-planer/this_is_rc_2016_web.pdf. Therein, some volunteers specialise in visiting people with dementia. The Norwegian Health Association, which is also the Norwegian Alzheimer’s Association, educates volunteers called “Aktivitetsvenn’ who focus on engage in activities with people who have dementia [36]. Norwegian Health Association. Aktivitetsvenn https://nasjonalforeningen.no/: Norwegian Health Association; 2023 [Available from: https://nasjonalforeningen.no/tilbud/aktivitetsvenn-for-personer-med-demens/.
